# Fatty acid metabolism of *Mycobacterium tuberculosis*: A double-edged sword

**DOI:** 10.15698/mic2022.05.777

**Published:** 2022-02-28

**Authors:** Camila G. Quinonez, Jae Jin Lee, Juhyeon Lim, Mark Odell, Christopher P. Lawson, Amarachukwu Anyogu, Saki Raheem, Hyungjin Eoh

**Affiliations:** 1 Department of Molecular Microbiology and Immunology, Keck School of Medicine, University of Southern California, Los Angeles, California, USA.; 2 Department of Life Sciences, Faculty of Science and Technology, University of Westminster, London, W1W 6UV, United Kingdom.; 3 School of Life Sciences, University of Lincoln, United Kingdom.; 4 Stratclyde Institute of Pharmacy and Biomedical Sciences, University of Strathclyde, Glasgow, United Kingdom.; 5 School of Biomedical Sciences, University of West London, London, United Kingdom.

**Keywords:** tuberculosis, metabolomics, drug tolerance, fatty acids, methylcitrate cycle

## Abstract

Unlike other heterotrophic bacteria, *Mycobacterium tuberculosis* (Mtb) can co-catabolize a range of carbon sources simultaneously. Evolution of Mtb within host nutrient environment allows Mtb to consume the host's fatty acids as a main carbon source during infection. The fatty acid-induced metabolic advantage greatly contributes to Mtb's pathogenicity and virulence. Thus, the identification of key enzymes involved in Mtb's fatty acid metabolism is urgently needed to aid new drug development. Two fatty acid metabolism enzymes, phosphoenolpyruvate carboxykinase (PEPCK) and isocitrate lyase (ICL) have been intensively studied as promising drug targets, but recently, Quinonez *et al.* (mBio, doi: 10.1128/mbio.03559-21) highlighted a link between the fatty acid-induced dormancy-like state and drug tolerance. Using metabolomics profiling of a PEPCK-deficient mutant, Quinonez *et al.* identified that over-accumulation of methylcitrate cycle (MCC) intermediates are phenotypically associated with enhanced drug tolerance against first- and second- line TB antibiotics. This finding was further corroborated by metabolomics and phenotypic characterization of Mtb mutants lacking either ICL or 2-methylcitrate dehydratase. Fatty acid metabolism induced drug-tolerance was also recapitulated in wildtype Mtb after treatment with authentic 2-methylisocitrate, an MCC intermediate. Together, the fatty acid-induced dormancy-like state and drug tolerance are attributed to dysregulated MCC activity.

## THE METABOLISM OF *M. TUBERCULOSIS*

Latent *M. tuberculosis* (Mtb) refers to a dormancy-like state in which Mtb is relatively less susceptible to a range of environmental stresses such as nutrient starvation and hypoxia, and antibiotic effects. To resist these challenges, Mtb exhibits metabolic plasticity that allows Mtb to phenotypically switch between replicating and nonreplicating states. Remarkably, Mtb survives lethal doses of mycobactericidal antibiotics by shifting its metabolic networks without genetic mutation-induced resistance. Therefore, understanding the intricate metabolic remodeling that Mtb uses to survive during infection and to form a dormancy-like state is critical for the development of new tuberculosis (TB) antibiotics. Quinonez *et al.* showed that suboptimal fatty acid metabolism of Mtb led to a drug-tolerant state. This fatty acid metabolism-induced drug-tolerance was metabolically associated with an accumulation of the methylcitrate cycle (MCC) intermediates, which was previously known to neutralize propionate toxicity. Three Mtb mutant strains lacking a gene in either gluconeogenesis or MCC were used to investigate the functional essentiality between fatty acid-induced drug tolerance and MCC intermediates.

## THE EFFECT OF FATTY ACID METABOLISM IN *M. TUBERCULOSIS* PEPCK-DEFICIENT STRAIN

Quinonez *et al.* established the metabolic state of PEPCK knockout strain (Δ*pckA*) cultured in either fatty acid or glycerol media. This strain lacks phosphoenolpyruvate carboxykinase (PEPCK), the first enzyme in gluconeogenesis that converts oxaloacetate to phosphoenolpyruvate (PEP). This reaction requires GTP as a cofactor. In fatty acid media, Δ*pckA* failed to grow but remained viable, whilst it grew at levels similar to that of wildtype in glycerol media. Targeted metabolomics of Δ*pckA* in acetate media showed that intermediates in the MCC accumulated (∼10 fold for 2-methylcitrate and 2-methylisocitrate) and the oxidative branch of the tricarboxylic acid (TCA) cycle (∼21 fold for aspartate, ∼17 fold for succinate, ∼58 fold for fumarate, and ∼69 fold for malate) as compared to those in glycerol media. qRT-PCR and ^13^C stable isotope tracing analyses confirmed that the accumulation of MCC and reductive TCA cycle branch intermediates were attributed to slowed TCA cycle activity, not to enhanced carbon fluxes.

In fatty acid media containing [U-^13^C] acetate, ^13^C stable isotope tracing analysis of Δ*pckA* further implied that Δ*pckA* may use preexisting carbon sources to fuel glycolysis. This was confirmed by the finding that Δ*pckA* sustained PEP abundance at a level similar to that of wildtype and its complement strain, however, the PEP biosynthesis occurred only within the unlabeled fraction. Given that Δ*pckA* lacks a gluconeogenesis carbon flux, the PEP levels could have only been sustained by the glycolytic activity from an endogenous carbon store. This metabolic remodeling is a likely mechanism underlying a dormancy-like state of Δ*pckA* in fatty acid media as glycolysis generates 15 times less ATP than the electron transport chain (ETC) activity mediated oxidative phosphorylation. Thus, Δ*pckA* maintained low levels of ATP due to low ETC and oxidative phosphorylation activity. Δ*pckA* in fatty acid media resists Bedaquiline (BDQ) treatment as this antibiotic kills Mtb by inhibiting its oxidative phosphorylation activity and ATP biosynthesis, which are not essential for Δ*pckA* viability in fatty acid media.

## DRUG TOLERANCE IN FATTY ACID-TRIGGERED DORMANT STATE

Mtb in a viable but reduced growth rate state is normally less susceptible to TB antibiotics. Δ*pckA* in fatty acid media resists first- and second-line TB antibiotics as fatty acid enhances the formation of viable but reduced growth rate population in Δ*pckA*. This was confirmed by enhanced persister formation (an isogenic drug-tolerant Mtb population) in Δ*pckA* in fatty acid media after treatment with *D*-cycloserine, an effective cell-lysing antibiotic, which was not observed in glycerol media. Indeed, the viability rate of Δ*pckA* in fatty acid media was significantly greater than that in glycerol media after isoniazid (INH) or BDQ treatment (∼13 fold (0.26% and 0.02%) or ∼487 fold (4.4% and 0.0009%)) respectively. Metabolomics of Δ*pckA* after antibiotic treatment confirmed the role of over-accumulated intermediates in MCC and reductive TCA cycle branch in resisting antibiotic treatment.

## ACCUMULATIN OF METHYLCITRATE CYCLE INTERMEDIATES AND DRUG TOLERANCE

To functionally link fatty acid-induced metabolic remodeling of Δ*pckA* to drug tolerance, two mutants lacking enzyme activity in the MCC were tested: an isocitrate lyase knockdown strain (ICL KD) and a 2-methylcitrate dehydratase knockout strain (Δ*prpD*). ICL catalyses the last step (converting 2-methylisocitrate to succinate and pyruvate) in the MCC, whilst PrpD catalytically converts 2-methyl-citrate to 2-methyl *cis*-aconitate, the second step in the MCC. As toxic propionyl-CoA is an initial substrate of the MCC, the growth kinetics of these two mutants were assessed in media containing propionate. Similar to the Δ*pckA* growth phenotype, ICL KD was unable to grow but a small fraction remained viable in propionate media due to over-accumulation of MCC intermediates. The growth rates and viability of Δ*prpD* were relatively unaltered in propionate media due to no quantitative change in MCC intermediates.

Upon INH treatment, the survival rate of ICL KD in propionate media was significantly greater by ∼7.0 fold as compared to that of wildtype and ∼2.8 fold as compared to that of Δ*prpD*. Metabolomics analysis indicated that ICL KD accumulated MCC intermediates but not reductive TCA cycle branch intermediates, suggesting a crucial role of MCC, but not TCA cycle, in resisting antibiotic effects. This was further confirmed by monitoring wildtype Mtb drug tolerance after treatment with MCC intermediate. Pre-treatment with 5 mM 2-methylisocitrate enhanced the levels of wildtype Mtb drug tolerance levels against INH or BDQ by ∼2.0 or ∼ 3.0 folds even in glycerol media as compared to those with no treatment.

## PROPOSED DRUG TOLERANCE MECHANISM OF Δ*pckA*

**Figure 1:**
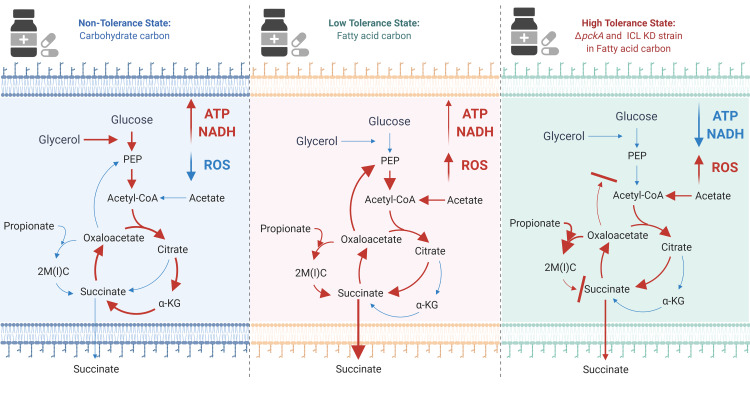
FIGURE 1: The effect of carbon metabolism on drug tolerance of Mycobacterium tuberculosis. The new findings indicate that fatty acids (acetate or propionate) alter Mtb central carbon metabolism, triggering a dormant-like state and drug-tolerance. In glycerol media, glycolysis and TCA cycle are active to biosynthesize ATP and NADH. NADH serves as a substrate of ETC activity, leading to biosynthesis of high levels of ATP (Left panel). Fatty acids activate the methylcitrate cycle (MCC) and accumulates the reductive TCA cycle branch intermediates. Accumulated MCC intermediates alter ETC activity and increases the ROS level as compared to those in glycerol media. Succinate secretion is an additional activity involved in maintaining the membrane potential (Middle panel). ΔpckA and ICL KD in fatty acid media induce metabolic remodeling associated with high drug tolerance. Over-accumulated MCC intermediates, slowed TCA cycle activity, reduced ATP levels, induced ROS, increased NADH/NAD ratios, and a relatively stable membrane potential are mechanistic bases underlying fatty acid-induced drug tolerance of ΔpckA and ICL KD (Right panel). Figures were created by BioRender.com.

Besides accumulated MCC intermediates, slowed TCA cycle activity, low levels of oxidative phosphorylation activity, and accompanying reduced ATP levels in acetate media, Δ*pckA* also demonstrated a high level of reactive oxygen species (ROS), increased NADH/NAD ratios, and a stable membrane potential as additional mechanistic bases underlying fatty acid metabolism induced drug tolerance. Upon antibiotic treatment, both ATP and ROS levels of Δ*pckA* were not as negatively impacted as those of wildtype, and the membrane potential was relatively unaffected by the INH treatment. A putative mechanism behind the membrane potential homeostasis is the succinate secretion as Δ*pckA* secreted succinate at a significantly greater level in acetate media than that of wildtype in acetate media or Δ*pckA* in glycerol media. Collectively, slowing down the TCA cycle activity and accumulating MCC intermediates caused a decrease in reducing equivalents (NADH and FADH_2_) required to sustain the ETC activity, leading to reduced NAD recycling, respiration, and intracellular ATP, ultimately leading to growth arrest and drug tolerance (**Figure 1**).

## FATTY ACID METABOLISM OF Mtb

The role of fatty acid metabolism of Mtb has been studied as a source of potential drug targets. In this study, Quinonez *et al.* investigated the understudied side effects arising from suboptimal regulation of fatty acid metabolism on Mtb growth phenotypes and drug tolerance. A drug discovery platform should acknowledge over-accumulated MCC intermediates as a metabolic cause to inversely invoke drug tolerance under a specific host nutrient environment.

